# Advance care planning dispositions: the relationship between knowledge and perception

**DOI:** 10.1186/s12877-019-1113-3

**Published:** 2019-04-24

**Authors:** Anne Cattagni Kleiner, Brigitte Santos-Eggimann, Sarah Fustinoni, Anne-Véronique Dürst, Katja Haunreiter, Eve Rubli-Truchard, Laurence Seematter-Bagnoud

**Affiliations:** 1 Center for Primary Care and Public Health (Unisanté), University of Lausanne, Route de la Corniche 10, 1010 Lausanne, Switzerland; 2 Service of geriatric medicine and geriatric rehabilitation, Lausanne university hospital, Chemin de Mont Paisible 16, 1011 Lausanne, Switzerland; 3 School of social and education studies (EESP), Higher school of social work and health, Chemin des Abeilles 14, 1010 Lausanne, Switzerland; 4 Chair of palliative geriatric care, Department of medicine, Lausanne university hospital, Av. Pierre Decker 5, 1011 Lausanne, Switzerland

**Keywords:** Advance care planning, Advance directives, Health care proxy, Knowledge, Perception

## Abstract

**Background:**

Legal dispositions for advance care planning (ACP) are available but used by a minority of older adults in Switzerland. Some studies found that knowledge of and perception of those dispositions are positively associated with their higher usage. The objective of the present study is to test the hypothesis of an association between increased knowledge of ACP dispositions and a more positive perception of them.

**Methods:**

Data collected in 2014 among 2125 Swiss community-dwellers aged 71 to 80 of the Lausanne cohort 65+ (Lc65+), a population-based longitudinal study on aging and frailty. Data collection was conducted through a questionnaire on knowledge, use and perception of lasting power of attorney, advance directives and designation of a health care proxy. Covariables were extracted from the Lc65+ database. Bivariable and multivariable regression analyses assessed the association between level of knowledge and perception.

**Results:**

Half the participants did not know about legal dispositions for ACP; filing rates were 14% for advance directives, 11% for health care proxy and 6% for lasting power of attorney. Level of knowledge about the dispositions was associated with a more positive perception of them, even when adjusting for confounding factors.

**Conclusion:**

Although the direction of the association’s causality needs more investigation, results indicate that better knowledge on ACP dispositions could improve the perception older people have of them. Communication on dispositions should take into account individual knowledge levels and address commonly enunciated barriers that seem to diminish with increased knowledge.

## Background

In 2013 Switzerland revised the National Adult Protection Law to better match the current societal trend towards the right of self-determination. Three legal advance care planning (ACP) dispositions are now available country-wide to allow the communication of personal wishes ahead of time in case of lost capacity for decision-making or for expressing oneself. Lasting power of attorney (LPOA) is a disposition that allows individuals to designate a trusted person for personal assistance and/or wealth management and/or legal representation. Advance directives (AD) can be drafted to specify which medical treatment one would or would not want to receive. Finally, there is the possibility of naming a health care proxy (HCP) who will take on the role of surrogate decision-maker when facing different treatment options if needed. LPOA and HCP both require the approval of the designated person. In Switzerland, not only an HCP but also a person designated by the LPOA can take on the role of surrogate medical decision-maker if needed and if the LPOA was previously defined as such. Also, advance directives can but do not always include the designation of a health care proxy. None of the LPOA, the AD or the designation of an HCP is mandatory. In the absence of any of those ACP dispositions, the law defines who shall take such responsibility for someone having lost decisional capacity (first any legal guardian, followed respectively by the spouse or registered partner if living together, a household member, descendants and parents, all on the condition that they were already providing regular personal help to this person).

Older adults are more directly concerned by legal dispositions for ACP but seldom use them. Local studies have focused on AD, probably because several Swiss states had already passed a law on AD in the late 1990s, while designation of an HCP is often embedded as a subsection of AD, and LPOA is a new disposition introduced in 2013 in the revised National Adult Protection Law. In 2009–2010 the proportion of persons with AD amounted to 25% among primary care patients aged 65 and over living in and around the Swiss town of Rorschach, and 34% among people 71 and over in a random sample of the general Swiss population [1, 2]. Even among patients facing life-threatening health problems, less than a third formally communicated their wishes (29% of Swiss palliative care patients) [3]. However, more recent national data indicate an increase in the AD completion rate from 25% in 2014 to 36% in 2017 among older adults [4]. Data are nevertheless geographically very different in Switzerland, with a much higher rate in German-speaking than in French or Italian-speaking regions of the country. In Vaud, a French-speaking state in which the present study took place, the Swiss Health Observatory reports a 2017 AD completion rate of 16%, which is much lower than the national average. These studies indicated lower completion rates in Switzerland as compared to North America, where 50 to 68% of individuals 65 and over reported having filed AD [5, 6]. Such a gap between Swiss and US rates might be explained by an earlier implementation of AD in the US, as shown by the fact that US figures increased from 47% of decedents with AD in 2000 to 72% in 2010 [7].

Data on reasons for infrequent use of legal dispositions for ACP outside of the end-of-life population are scarce and mainly focused on AD. Studies found that lack of knowledge was the primary or one of the primary reasons why AD were not initiated [5, 8, 9]. The conjunction of a frequent lack of knowledge of the ACP dispositions and an equally frequent reported willingness to use them when informed of their existence [10, 11] points to information as a key determinant of completion. Other studies revealed more positive perceptions towards dispositions among people who had completed them than among people who had not [11–14]. However, as far as we are aware, to date no studies have yet taken into account the association between knowledge and perception of legal dispositions for ACP.

The survey we conducted among community-dwelling older adults in Switzerland examining their knowledge, use, and perception of the three legal dispositions [10] allows for further understanding of the processes at work by investigating the association between knowledge and perception of each dispositions. The current work is based on Fried’s demonstration that Prochaska and Velicer’s transtheoretical model of health behavior change (TTM) [15] can be applied to advance care planning [16]. Fried used the TTM model to develop personalized health messages to promote engagement in advance care planning [17]. The TTM states that “one has to go through different stages of readiness to change (precontemplation, contemplation, preparation, action and maintenance) and that health promotion messages should be tailored to those different stages to be efficient” [15]. Prochaska and Velicer, as well as Fried, have studied the pattern of the decisional balance for engaging in various behaviors at each of these stages, i.e. the proportion of the pros and cons at each stage. Both systematically found that in order to progress from precontemplation (no intention to take action because of lack of will or lack of knowledge) to the contemplation stage (intention to change in the next 6 months), the pros of changing must increase, and that to progress from the contemplation to action stage (overt changes made in the past 6 months), the cons of changing must decrease to reach a lower proportion than the pros [15, 16]. Based on the premise that there exists a maturation process before the completion of a disposition, and that each stage of this process needs to be identified, the current study investigated the relationship between knowledge and perception of each of the dispositions. Our data, by including three levels of knowledge (knowledge, partial knowledge and no knowledge prior to the survey) and gathering information about perceived advantages and disadvantages of each of them, allowed us to test the following hypotheses:

1. For each disposition, individuals who were aware of it prior to the study should report a more positive perception, by selecting advantages more often and disadvantages less often than people who did not know about it. This association should persist after adjustment for potential confounding factors.
2. People who knew about the dispositions should also report a more positive perception of them than the individuals who only had partial knowledge of them.

Testing these hypotheses will provide additional insight into the mechanisms involved in the decision to file or not dispositions regarding advance care planning, thus allowing better communication strategies on these issues.

## Methods

### Data source and participants

Data were drawn from a 2014 survey of 2125 older residents in the city of Lausanne, Switzerland, on the new National Adult Protection Law. This survey was integrated into the Lausanne cohort Lc65+, a longitudinal study of randomly selected older people living in the community since 2004 [18]. A month after the regular annual questionnaire was received, the survey was sent to wave 1 and wave 2 participants of the Lc65+ study’s 2014 follow-up, i.e., all between the ages of 71 and 80.

### Data collection

The self-administered anonymous 4-page paper questionnaire was developed by an interdisciplinary team composed of a geriatrician, a psychologist, a law professor and a hospital chaplain, addressing end-of-life issues in their different fields of expertise, in collaboration with the Lc65+ study team. Content validity was assessed within the same team and the questionnaire was not submitted to outside experts.

The questionnaire laid out objectives, content, and different modalities of the three dispositions before asking participants about their previous knowledge, use, and perception of them. AD writing and the designation of an HCP were treated as separate outcomes since it is possible to opt for one disposition without opting for the other. Knowledge about advance care planning dispositions was addressed by asking participants whether they previously knew about LPOA, then about AD, and finally about the possibility of naming an HCP. Response options for each of these questions were “yes”, “partially”, and “no”.

**Fig. 1:**
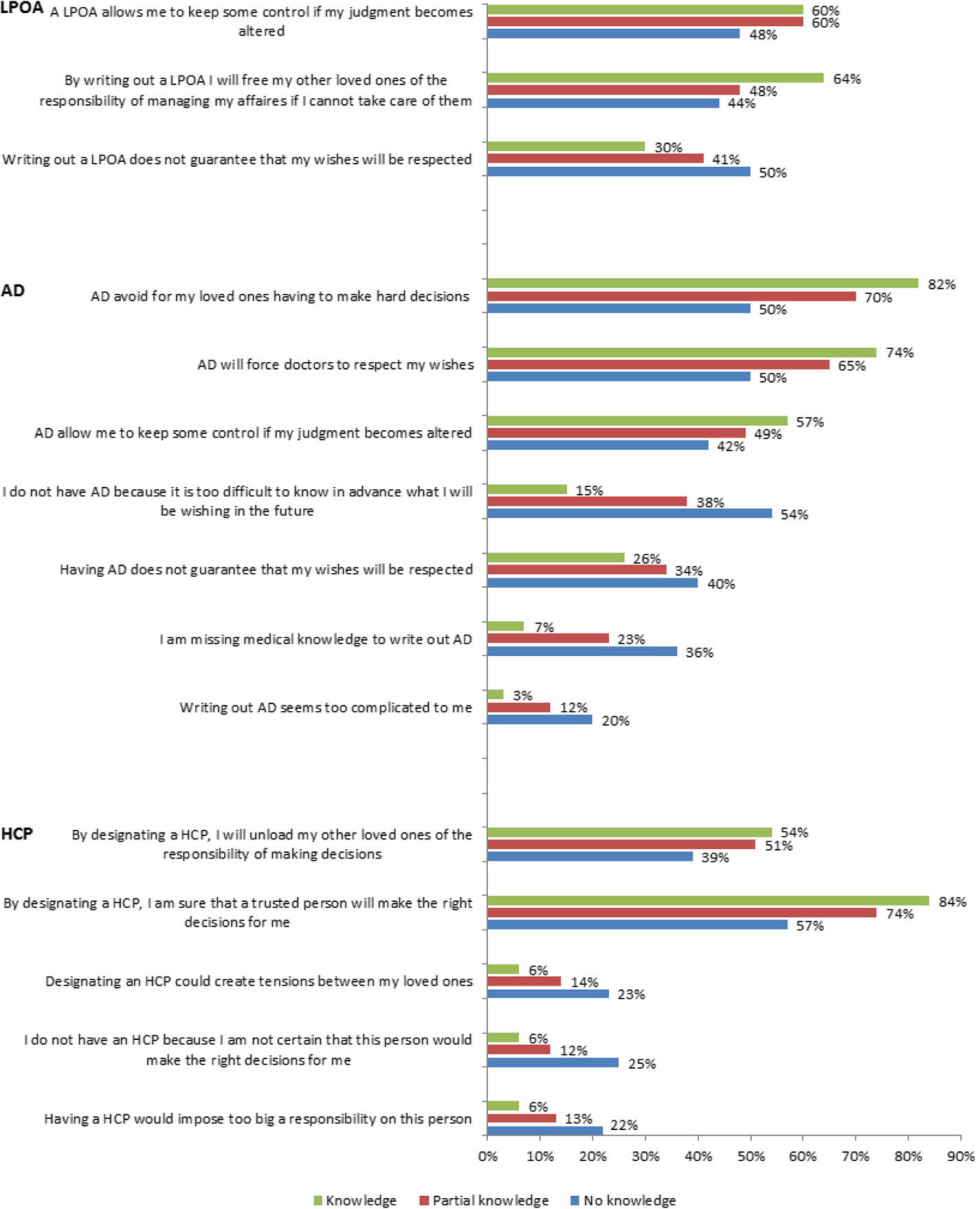
Percentage of individuals selecting each statement, by level of knowledge of ACP dispositions. Note: Chi square *p* values < 0.001 for all comparisons

**Fig. 2:**
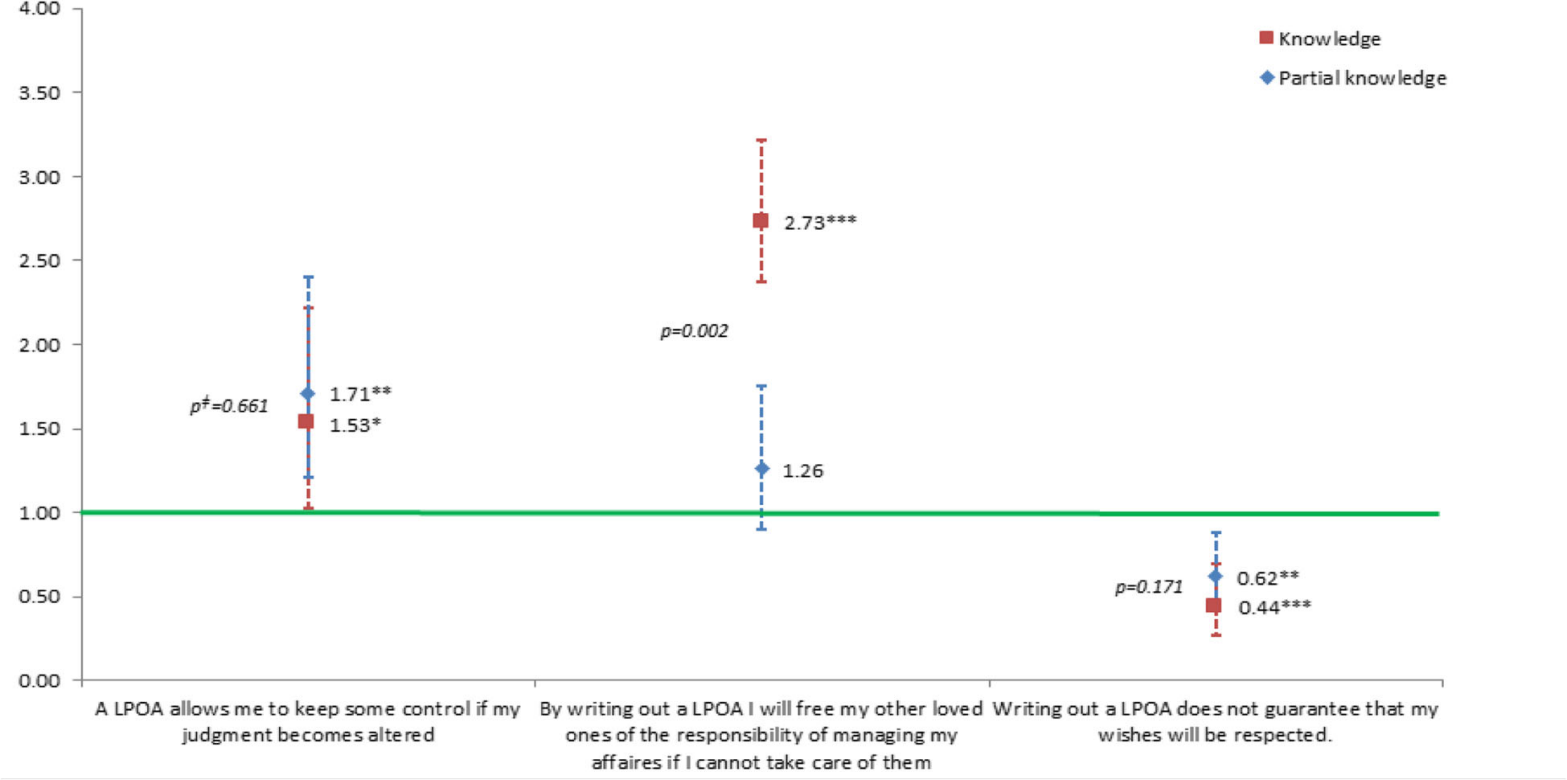
Associations^ƚ^ between agreeing with diverse statements regarding lasting power of attorney (LPOA) and level of knowledge about this disposition (OR and 95% CI, reference: no knowledge). ^ƚ^Odd ratios from multivariable regression analysis adjusted for the following covariables: gender; age group; educational level; living arrangement; number of children; born in the country; financial status; self-rated health; functional status; memory or concentration impairments; depression or anxiety symptoms; number of self-reported active chronic diseases; fear for own health; in the past 5 years: death of a loved one, difficulty in obtaining professional help or care or becoming a caretaker for a loved one, serious illness/accidents oneself/loved one, none of the events above; importance of spirituality, and communication with doctors. ^ǂ^Wald test’s *p* value of the difference between ORs (knowledge – partial knowledge). *p < 0.05, **p < 0.01, ***p < 0.001

**Fig. 3:**
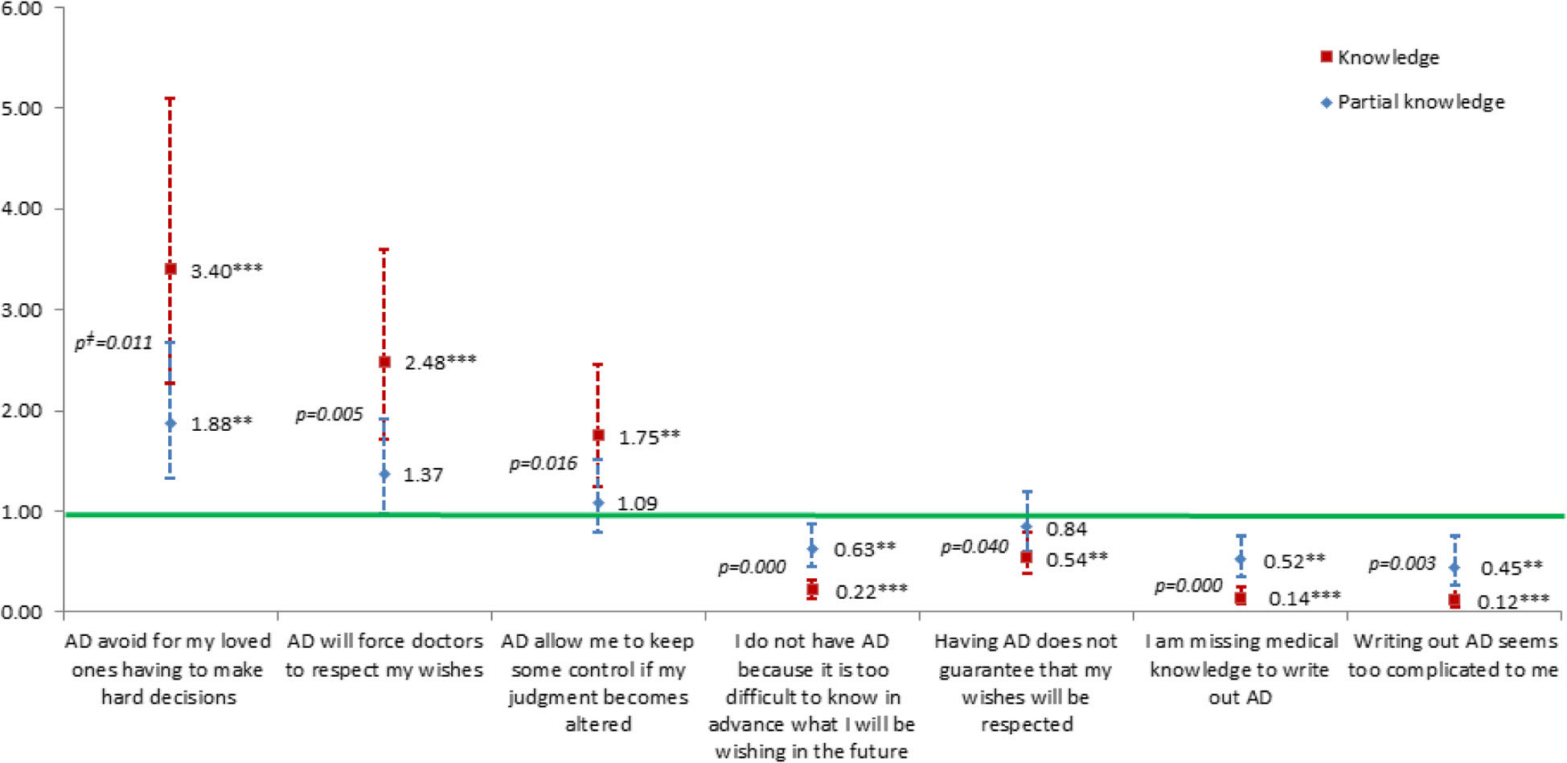
Associations^ƚ^ between agreeing with diverse statements regarding advance directives (AD) and level of knowledge about this disposition (OR and 95% CI, reference: no knowledge). ^ƚ^Odd ratios from multivariable regression analysis adjusted for the following covariables: gender; age group; educational level; living arrangement; number of children; born in the country; financial status; self-rated health; functional status; memory or concentration impairments; depression or anxiety symptoms; number of self-reported active chronic diseases; fear for own health; in the past 5 years: death of a loved one, difficulty in obtaining professional help or care or becoming a caretaker for a loved one, serious illness/accidents oneself/loved one, none of the events above; importance of spirituality, and communication with doctors. ^ǂ^Wald test’s p value of the difference between ORs (knowledge – partial knowledge). *p < 0.05, **p < 0.01, ***p < 0.001

**Fig. 4:**
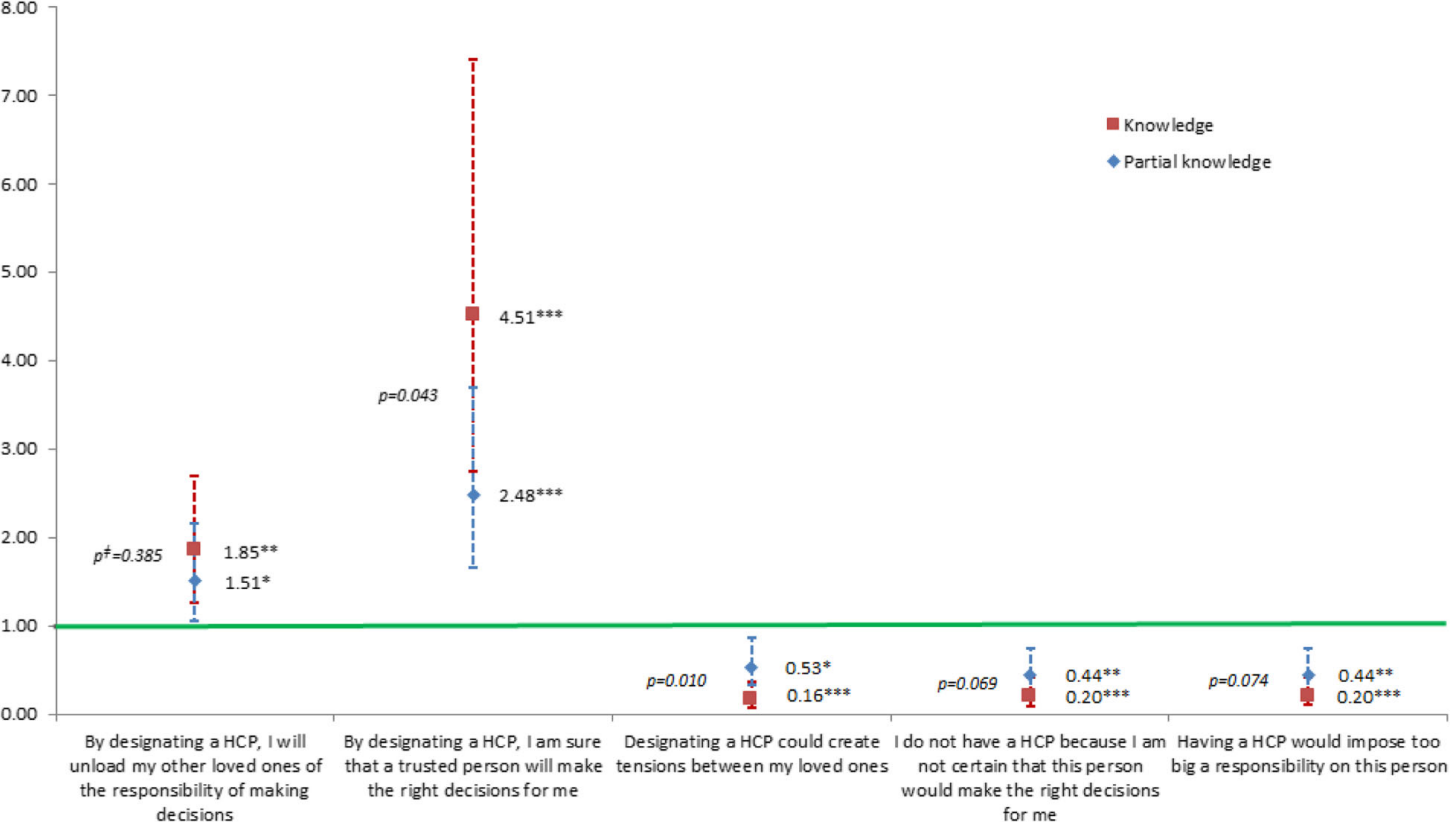
Associations^ƚ^ between agreeing with diverse statements regarding having a health care proxy (HCP) and level of knowledge about this disposition (OR and 95% CI, reference: no knowledge). ^ƚ^Odd ratios from multivariable regression analysis adjusted for the following covariables: gender; age group; educational level; living arrangement; number of children; born in the country; financial status; self-rated health; functional status; memory or concentration impairments; depression or anxiety symptoms; number of self-reported active chronic diseases; fear for own health; in the past 5 years: death of a loved one, difficulty in obtaining professional help or care or becoming a caretaker for a loved one, serious illness/accidents oneself/loved one, none of the events above; importance of spirituality, and communication with doctors. ^ǂ^Wald test’s p value of the difference between ORs (knowledge – partial knowledge).. *p < 0.05, **p < 0.01, ***p < 0.001

Perception about each disposition was captured by asking participants to select statements with which they were in agreement among lists of advantages and disadvantages (see Figs. 1, 2, 3 and 4 for a list of these statements).

The overall response rate was 80% (1701 valid questionnaires received), with slightly lower participation among women, first-wave individuals (76–80 years old) and people with lower educational attainment (under high school graduation). However, there was no gap larger than 3 percentage points between any subgroup response rate and the overall response rate. Item non-response ranged from 3 to 14%, and there was no imputation. Survey data were then merged with data collected through the Lc65+ annual questionnaires.

### Covariables

Most covariables described below were not collected with the survey, but during the annual Lc65+ study data collections.

The socio-demographic covariables were gender, age group (71–75 years-old vs 76–80 years-old), educational level (less than high school vs high school graduation and above), living arrangement (living alone or not), number of children (0, 1, 2 or more), place of birth (Switzerland vs other), and financial status (means-tested government subsidy recipient vs non-recipient).

Health status indicators were used. Participants were asked to self-rate their health (very good or good vs average to very bad). Functional status was assessed by asking about impairments in basic or instrumental activities of daily living (ADL) (no impairments vs at least 1 impairment) [19, 20]. Identification of memory and concentration impairments (0, 1, 2 or more) was based on the answers to a list of nine items, such as difficulty remembering conversations after a few days or difficulty learning how to use a new gadget or equipment in the home. Response options were “never,” “rarely,” “sometimes,” “often,” and “very often”, with the latter two considered in the identification of a memory or concentration impairment. The covariable depression or anxiety symptoms was based on three questions about sadness, anxiety, or lack of interest during the past 4 weeks. A positive answer to at least one of those three questions was coded as depression or anxiety symptom (yes vs no). Participants were asked to report chronic diseases they were suffering from in the past 12 months and the types of medications taken at least once a week. This information allowed for an estimation of the number (0, 1, 2 or more) of types of chronic conditions such as hypertension, hypercholesterolemia, cardiac disease, diabetes, pulmonary disease, stroke, cancer, Parkinson’s disease, Alzheimer’s disease, and HIV infection. The number of hospital stays during the past 5 years (0, 1, 2 or more) was also used as a covariable.

Other personal characteristics were used in the analyses, mainly related to experience, such as worrying about one’s own health (very or quite worried, a little or slightly, not at all worried) and potentially stressful events (yes vs no) having occurred in the 5 years preceding the survey (see Table 1 for the list of events). The covariable “none of the events above” was built afterward. The covariable labeled “importance of spirituality” when facing stressful events (moderate to great importance vs little or no importance) was based on the question “When facing stressful events, what is the importance of spirituality, religion, or philosophical thinking?” The last covariable “communication with doctors” (optimal vs non-optimal) combined the responses to a set of five questions: feeling of being listened to during visits, of receiving enough information about treatment options, that one’s own emotional and psychological needs were taken into account, that preferences regarding treatment options were taken into account, and that doctors know precisely about one’s own living conditions (family, housing, activities, etc.). Response options for those five items were “yes, totally”, “rather yes”, “rather no” and “not at all”. When all five responses were “yes, totally”, the response option was coded as “optimal”; otherwise, it was coded as “non-optimal”.

### Data analysis

Bivariable analyses, using Pearson’s Chi square test, compared the proportion of individuals having selected each advantage or disadvantage about the dispositions, according to their level of knowledge about them. Results were considered significant when the *p*-value was less than 0.05.

Fifteen multivariable logistic regression analyses were performed, each using one of the proposed advantages or disadvantages as the outcome and the level of knowledge as the explanatory variable. Covariables were included in the regression analyses if they were significantly correlated with checking at least one of the fifteen statements (Pearson’s Chi square test, p-value < 0.05). Variables that were not included in the regression analyses because they did not meet this condition were “number of hospital stays in the past five years”, “conflict within the family in the past five years”, and “none of the events above”. For each multivariable model, a Wald test was computed to check for a significant difference in estimated odds ratios for knowing versus partially knowing about the disposition, taking “no knowledge” as the reference category. All analyses were conducted using Stata 13 software (StataCorp, College Station, TX).

## Results

### Description of the sample

Table 1 provides a description of the socio-demographic characteristics, health status indicators, and other personal characteristics of the sample. A higher proportion of individuals were female and belonged to the younger age group. One out of five participants did not have any children, one out of four was born outside of the country, more than a half did not have a high school degree, and about one out of six respondents declared receiving means-tested government subsidies. Over two-thirds reported being in good or very good health and not having any memory or concentration impairment, while a third had anxiety or depression symptoms. Proportions of individuals with and without functional impairment were virtually equal. About three out of ten persons reported no chronic disease (of the options provided), and one half had been hospitalized in the 5 years prior to the survey. Half of the sample reported that they worried about their health, and the vast majority had experienced at least one stressful events in the past 5 years. Six out of ten people reported a moderate or high importance of spirituality when experiencing stressful events. About a third gave the maximum rating to describe the communication with their physician.

**Table 1:** Distribution of the study population by socio-demographic, health status and other personal characteristics

Characteristics	% (*N* = 1701)
Total	100
Gender *(n = 1701)*	
Male	40
Female	60
Age group *(n = 1701)*	
76–80 years	44
71–75 years	56
Educational level *(n = 1699)*	
Under high school graduation	58
Higer school graduation and higher	42
Living arrangment *(n = 1699)*	
Alone	44
Not alone	56
Number of children *(n = 1685)*	
0	21
1	16
2 or more	63
Born in the country *(n = 1698)*	
Yes	76
No	24
Financial status *(n = 1681)*	
Government subsidy recipient	16
Non-recipient	84
Self-rated health *(n = 1700)*	
Very good or good	68
Average to very bad	32
Functional status *(n = 1637)*	
No impairment with ADL	49
1 impairment or more	51
Memory or concentration impairments *(n = 1663)*	
0	69
1	16
2 or more	15
Depression or anxiety symptoms *(n = 1698)*	
No	67
Yes	33
Number of self-reported active chronic diseases *(n = 1693)*	
0	27
1	32
2 or more	42
Number of hospital stays in the last 5 years *(n = 1278)*	
0	49
1	24
2 or more	27
Worry about own health *(n = 1701)*	
Very or quite worried	33
A little or slightly worried	61
Not at all worried	7
Potentially stressful events in the past 5 years	
Death of a loved one *(n = 1651)*	59
Difficulty in obtaining professional help or care or becoming a caretaker for a loved one *(n = 1624)*	28
Serious illness/accidents oneself/loved one *(n = 1678)*	81
Conflict within the family *(n = 1638)*	31
None of the events above *(n = 1691)*	7
Importance of spirituality *(n = 1686)*	
Little or no importance	39
Moderate to great importance	61
Communication with doctors *(n = 1408)*	
Optimal	30
Non-optimal	70

### Knowledge about the legal dispositions for ACP

Almost half (47%; data not shown) of the participants were unaware of any of the three legal dispositions for ACP; 68% did not know about LPOA, 55% about AD, and 66% about the possibility of naming an HCP. The proportion of individuals knowing or partially knowing about AD or HCP was about the same, but more knew only partially about LPOA (21% vs 12% knowing about it) (data not shown).

### Completion of the legal dispositions for ACP

Overall, 14% of participants had completed or were in the process of completing AD. This proportion increased to 32% among participants who knew about AD prior to the survey. A lower completion rate was observed regarding LPOA (6% overall and 19% based on the individuals with at least some prior knowledge of it), while respectively 11 and 34% had or were in the process of selecting an HCP (data not shown). Among people who had not completed AD, 74% of individuals who knew about them prior to the survey, 64% of individuals with partial knowledge, and 44% with no knowledge declared that they would consider doing so. The same question regarding the LPOA showed that respectively 58, 59, and 44% would look into it (data not shown).

### Perception of the legal dispositions for ACP

Proportions of individuals selecting each proposed advantage and disadvantage are presented in Fig. 1. There was a positive association between pre-survey knowledge of the dispositions and a more positive perception, even when adjusting for socio-demographic or personal characteristics and health status indicators (Figs. 2, 3 and 4). Indeed, among people who knew about those dispositions before the survey, the odds of selecting any positive statement were higher, and the odds of selecting any negative statement were lower.

Furthermore, Figs. 2, 3 and 4 show that when comparing people having declared that they knew about the dispositions with people only having a partial knowledge of them, the odds were systematically stronger for the positive statements and weaker for the negative statements. This trend appeared clearly for all but one of the dispositions and statements (Figs. 2, 3 and 4). These differences were all statistically significant when pertaining to statements about advance directives (Fig. 3)

.

## Discussion

This study collected novel data on the knowledge and perception of three dispositions allowing communication in advance of one’s own wishes about medical and administrative matters. These results are based on a large representative sample of older Swiss community-dwellers, with covariables related to many sociodemographic, health status, and other personal characteristics. Its first finding was that the majority of the population aged 71–80 living at home did not know about the dispositions. A lower proportion of people did not know about advance directives compared to HCP or LPOA. Also, compared with AD or HCP, partial knowledge was most prevalent for LPOA. Both observations were probably due, at least in part, to the newness of LPOA and to the fact that the designation of an HCP can be included in AD. Then, similar to previous observations [11], a high proportion of people without any previously filed dispositions considered filing them, showing that there is a real interest provided that people are aware of those possibilities.

Among the younger part of the older population in this study, the AD completion rate (14%) and the proportion of individuals having named an HCP (11%) are much lower than those observed in the United States among older adults living in the community [21–23]. However, AD seem to be more frequent in Switzerland than in most other European countries [24, 25], and it is expected that the federal law that has come into force in 2013 will give them, along with the LPOA and the possibility of naming an HCP, more visibility for the public and for health practitioners.

This study showed that, whatever the direction of the causality, there is a solid association between knowledge and perception of the dispositions, that this association is a positive one, and that it gets stronger with an increased level of knowledge. Results even went beyond what was laid out in our hypotheses. We were expecting in general a more positive perception when comparing people who knew about the dispositions before the survey with people who did not know about them. Bivariable and multivariable analyses adjusting for a large range of covariables proved this true and statistically significant for every single one of the 15 arguments in favor and against filing dispositions. Furthermore, strikingly, the strength of the association between knowledge and perception was greater with an increased level of knowledge for virtually each proposed statement. There was a very clear trend toward a more positive perception with a higher level of knowledge. Among the perceptions particularly concerned by that effect were some barriers commonly found in the literature: the difficulty to project oneself [26, 27] and the feeling of lacking skills to draft AD (medical knowledge, ease with writing such documents) [1]. Gaps between these two levels of knowledge were also very noticeable with the selection of advantages and disadvantages pertaining to concerns for loved ones in general: nominating an HCP was more often viewed as a risk for creating tensions between loved ones when individuals only had a partial knowledge about it, while the group with knowledge thought more often that having AD would avoid loved ones having to make hard decisions. Another more noticeable difference between the two levels of knowledge lays with the argument that “by designating an HCP, I am sure that a trusted person will make the right decisions for me”, which was much more approved of with knowledge compared with partial knowledge.

### Practice implications

Because of the cross-sectional nature of the data, determining the causality of the positive association between level of knowledge and positive perception of the dispositions was not possible. However, it seems likely that knowledge and perception influence each other, and that their relationship is not unilateral. Searching for strategies for improving both is important. More specifically, the fact that this relationship was particularly salient in commonly enunciated barriers to their use implies that communication about dispositions might have a major effect on the way people perceive them.

As previously discussed, our data pointed to the relationship between a low level of knowledge and the perceived difficulty of deciding now for the future as a barrier to write AD. These results indicate the need for specifying that they can and even probably should be regularly reviewed and if necessary, revised. Another argument for encouraging regular updating of AD is that preferences do not always remain stable over time, even over a short time span [28, 29]. Finally, it would be useful that the communication about ACP dispositions specify that they are reversible if the capacity of judgment is recovered and, therefore, could be only temporary.

Attention should be paid to the vocabulary used when communicating about AD and LPOA, but also to the one employed in pre-prepared forms. Furthermore familiarity with specialized terms might differ from one socioeconomic group to another [30].

Results show that the perception concerning the effects that filing AD or designating an HCP could have on loved ones is significantly associated with the level of knowledge. Effective communication should stress the fact that in the absence of such dispositions, loved ones will be asked to play a role anyway, for which they might not be prepared. Beside the responsibility of the task, focus should also be put on the likely legitimizing effect of advance care planning dispositions for the proxy [31].

AD and the designation of an HCP are often discussed together in brochures, and LPOA are discussed separately. Fried et al. found that the completion of a living will was associated with the completion of other non-health-related end-of-life preparing activities, suggesting that completion of AD could be promoted in real estate planning for example [32]. This is also relevant in the Swiss context. We therefore propose that during communication campaigns, all these aspects be addressed in the same document.

Parallel to our findings that a process of maturation seems necessary and that increased knowledge would probably feed this process, Ramsaroop, Reid, and Adelman [33] observed that the most successful interventions designed to increase AD completion rates were those involving repetitive patient-health care professional interactions. Others have found that most older adults were willing to talk with their physician about end-of-life decisions, and that a significant proportion thought that these discussions should be initiated by the physicians [22, 23, 34]. However, 2017 national data show that only 5.9% of people aged 65 and older had had a discussion on this topic with their regular physician [4].These findings should encourage primary care physicians to initiate advance care planning discussions with their patients. To help trigger the maturation process, and in line with Fried et al’s having developed different brochures tailored to individuals at various stage of a change process [17], we could imagine a toolkit for health practitioners helping them to assess their patients’ level of knowledge about the disposition, in order to give them relevant information. Level of knowledge would here be used as a practical proxy for stage of change that can quickly be assessed by simply asking about it.

### Limitations

As mentioned before, longitudinal data would have been a true asset, allowing the investigation of causality in the knowledge-perception association. Additionally, because this sample included only 71–80 year-old people living at home, the study results cannot be generalized to all older community-dwellers in Switzerland. Finally, the questionnaire was developed and evaluated for its validity by an interdisciplinary team of professionals dealing with end-of-life issues in their different fields of expertise. However, it was not submitted to an outside expert panel for review and did not go through a face validity test with the population of interest.

## Conclusion

While filing advance care planning dispositions is not mandatory, and whether one should do so is a question still open for debate, everyone should at least know about them in order to make an informed decision. Our results suggest that stakeholders promoting such dispositions should communicate about them as early as possible, in order to increase the level of knowledge and thus encourage a more positive perception. Giving information early in life would also benefit to people in a caregiving role. Finally, gathering longitudinal data would help to assess the best time to present the information about the dispositions. Results show that there is still great room for improvement in the way information about the dispositions is provided, and point to the necessity of designing communications campaigns allowing a progressive understanding of these dispositions.

## Abbreviations

ACP: Advance care planning
AD: Advance directives
HCP: Health Care Proxy
LPOA: Lasting power of attorney
